# College and Hospital Notes

**Published:** 1899-06

**Authors:** 


### COLLEGE AND HOSPITAL NOTES.

The University and Bellevue Hospital Medical College held its annual commencement exercises May 16, 1899, at the Metropolitan Opera House, New York, at 8 p. m., at which time 162 graduates received their degrees. Of this number twenty-nine secured hospital appointments after competitive examinations.

By the will of the late Dr. Valentine Mott, perpetual provision was made for the following medals : A gold medal to the candidate who shall prepare the best anatomical or anatomico-surgical preparation· A silver medal to the second best of that description. A bronze medal to the candidate who shall furnish the best book of recorded cases and remarks of the professor at the surgical clinics. The gold medal is awarded to Albert S. Morrow, A. B., class 1901. The silver medal is awarded to Arthur B. Bradshaw, class 1901. The bronze medal is awarded to Willard Monfort, class 1899.

